# Optical Gas-Cell Dynamic Adsorption in a Photoacoustic Spectroscopy-Based SOF_2_ and SO_2_F_2_ Gas Sensor

**DOI:** 10.3390/s22207949

**Published:** 2022-10-18

**Authors:** Ying Zhang, Mingwei Wang, Pengcheng Yu, Zhe Liu

**Affiliations:** Scientific Research Institute of Electric Power, Guizhou Power Grid Company Ltd., 251 Jiefang Road Nanming District, Guiyang 550002, China

**Keywords:** optical gas sensing, photoacoustic spectroscopy, harmonic detection, photoacoustic cell, adsorption effect

## Abstract

SO_2_F_2_ and SOF_2_ are the main components from the decomposition of insulation gas SF_6_. Photoacoustic spectroscopy (PAS) has been acknowledged as an accurate sensing technique. Polar material adsorption for SO_2_F_2_ and SOF_2_ in the photoacoustic gas cell of PAS may affect detection efficiency. In this paper, the optical gas-cell dynamic adsorptions of four different materials and the detection effects on SO_2_F_2_ and SOF_2_ are theoretically analyzed and experimentally demonstrated. The materials, including grade 304 stainless steel (SUS304), grade 6061 aluminum alloy (Al6061), polyvinylidene difluoride (PVDC), and polytetrafluoroethylene (PTFE), were applied inside the optical gas cell. The results show that, compared with metallic SUS304 and Al6061, plastic PVDC and PTFE would reduce the gas adsorption of SO_2_F_2_ and SOF_2_ by 10 to 20% and shorten the response time during gas exchange. The complete gas defusing period in the experiment was about 30 s. The maximum variations of the 90% rising time between the different adsorption materials were approximately 3 s for SO_2_F_2_ and 6 s for SOF_2_, while the generated photoacoustic magnitudes were identical. This paper explored the material selection for PAS-based gas sensing in practical applications.

## 1. Introduction

SF_6_ is widely used in gas-insulated switchgear (GIS), with excellent insulation and arc extinguishing properties. Due to partial discharge and overheating, SF_6_ will decompose and cause an insulation breakdown in GIS, leading to equipment failure [1]. Therefore, it is important to monitor the decomposition state of SF_6_ in GIS. SO_2_F_2_ and SOF_2_ are the main substances from the decomposition of SF_6_ [2]. Versatile sensing techniques for SO_2_F_2_ and SOF_2_ have been reported and improved. Infrared absorption spectroscopy is one of the popular approaches, with features of high accuracy, rapid response, selectivity, etc. In 1997, Heise et al. used Fourier transform infrared spectroscopy to monitor the decomposition of SF_6_ under discharge conditions and obtained the products of CF_4_, SOF_2_, SO_2_F_2_, and SO_2_ with ppm-level accuracy [3]. Tunable diode laser absorption spectroscopy (TDLAS) has also been investigated for ultra-sensitive gas detection [4]. An extended optical gas chamber could be realized by hollow-core optical fibers to achieve ppb-level sensitivity in TDLAS [5,6,7].

Recently, photoacoustic spectroscopy (PAS) has also become a hot topic, due to its high selectivity and sensitivity, as well as its fast response [8,9]. It has been used to detect H_2_S, SO_2_, CO, and other substances from SF_6_ decompositions with sub-ppm detection limits [10,11,12]. Nevertheless, few works of literature have reported on the PAS-based detection of SO_2_F_2_ and SOF_2_. Both SO_2_F_2_ and SOF_2_ are typical polar molecules that exhibit substance solubility and diffusion in polar solvents. Hence, in practical PAS implementations, it is necessary to analyze the adsorption between gas molecules and photoacoustic cells (PACs), which may influence the sensing performance.

In this paper, we analyzed and experimentally demonstrated the optical gas-cell dynamic adsorption with different materials in the PAS-based SOF_2_ and SO_2_F_2_ gas sensors. An H-type resonant PAC was designed and fabricated in this experiment. Adsorption mechanisms between gas molecules and four materials, i.e., grade 304 stainless steel (SUS304), grade 6061 aluminum alloy (Al6061), polyvinylidene difluoride (PVDC), and polytetrafluoroethylene (PTFE), were analyzed and discussed. Dynamic gas flow and concentration diffusing inside the four PACs were simulated and compared. The PAS-based gas sensor was established, and four PACs of different surface materials were fabricated. Compared with the metallic SUS304 and Al6061, the plastic PVDF and PTFE could reduce the gas adsorption behavior and shorten the response time. The complete gas-diffusing period was approximately 30 s in the experiment. The maximum variations of the 90% rising time between the different adsorption materials were approximately 3 s for SO_2_F_2_ and 6 s for SOF_2_, while the generated photoacoustic magnitudes were identical. The exchange variances of the gas concentrations between the metals (SUS304 and Al6061) and the plastics (PVDF and PTFE) were about 10% for SO_2_F_2_ and 20% for SOF_2_ in the employed PAS system.

This work provides guidance for the material selection of the optical gas cell in practical PAS sensing applications for polar gases.

## 2. Photoacoustic Spectroscopy Principle

The basic principle of PAS contains Beer–Lambert’s law and the photoacoustic effect. Beer–Lambert’s law describes the absorption of light by gas molecules. When light passes through gas, if the absorption spectrum of the gas overlaps with the optical wavelength, the output light intensity will decrease after the gas absorption. The decrescent is related to the gas absorption coefficient, the gas concentration, and the absorption optical path [13]:

(1) $$I\left(\lambda \right)=I_{0}\left(\lambda \right)exp\left[-\alpha \left(\lambda \right)CL\right]$$

where *λ* is the wavelength of the light, *I(λ)* is the output light intensity, *I_0_(λ)* is the input light intensity, *α(λ)* is the absorption coefficient of the gas, *C* is the concentration of the target gas, and *L* is the absorption optical path.

In the photoacoustic effect, when the intensity of the incident light is regularly modulated, the gas pressure changes regularly, forming a photo-generated acoustic pressure signal. The intensity, *S_PA_*, of the photoacoustic signal can be simply expressed as [14,15]:

(2) $$S_{PA}=K_{cell}\alpha CP_{0}$$

where *K_cell_* is the cell constant of the PAC, determined by the quality factor and volume of the PAC, as well as by the modulation frequency. *P_0_* is the optical power of the incident light. This indicates that when the gas cell, the light source, and the gas molecules are fixed, the intensity of the photoacoustic signal is proportional to the gas concentration.

## 3. Analysis and Simulation

### 3.1. Gas-Cell Design and Simulation

**Figure 1:**
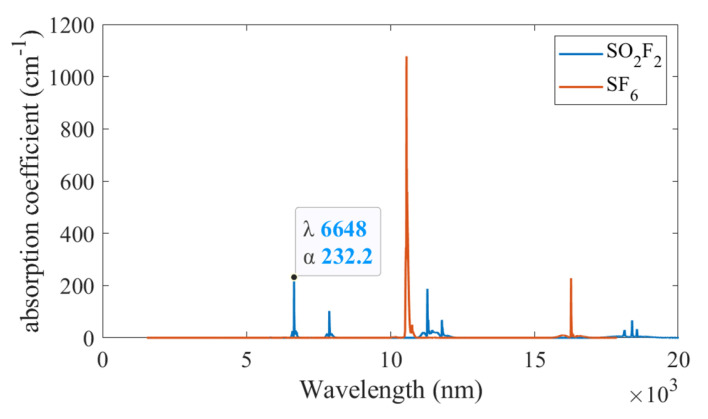
Absorption spectra of SO_2_F_2_ and SF_6_. The selected absorption line of SO_2_F_2_ is labeled at 6648 nm.

The amplitude of the acoustic signal is proportional to the absorption coefficient of the target gas. Figure 1 shows the absorption spectrum lines of SO_2_F_2_ and SF_6_ at the temperature of 25 °C and pressure of 1 atm, obtained from the acknowledged HITRAN database. The selected absorption line of SO_2_F_2_ is located at 6648 nm and has little cross interference with SF_6_. As found in the literature, the absorption line of SOF_2_ is located at approximately 7463 nm [16].

**Figure 2:**
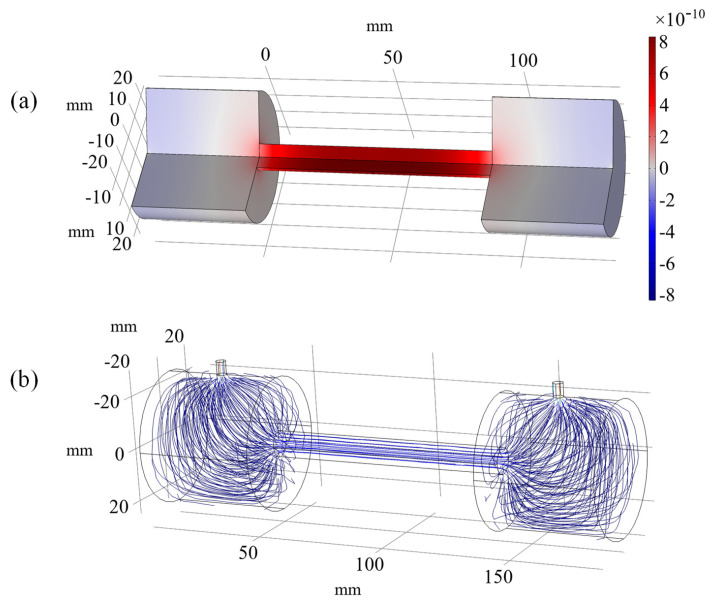
(**a**) The acoustic field and (**b**) the airflow distribution inside the PAC.

In the PAC, the photoacoustic effect occurs and the acoustic signal is excited. In this work, an H-type longitudinal resonant PAC was designed, with an acoustic resonator of 90 mm in length and 5 mm in radius, as well as symmetrical buffer volumes of 45 mm in length and 25 mm in radius. Figure 2a shows the simulated acoustic field distribution inside the gas cell, exhibiting its maximum point located at the center of the acoustic resonator. The simulation is based on the finite element method and realized using the COMSOL Multiphysics software package. In particular, the thermoviscous acoustics model and the pressure acoustics model were employed for the photoacoustic simulation [17].

To analyze the adsorption effect and the gas exchange during the entire ventilation process, the dynamic airflow inside the PAC was further simulated, as shown in Figure 2b. It was found that the airflow inside the PAC was not homogenously distributed during ventilation, while the airflow inside the central cylindrical resonator was rather uniform.

### 3.2. Gas Molecule and Adsorption Material

**Figure 3:**
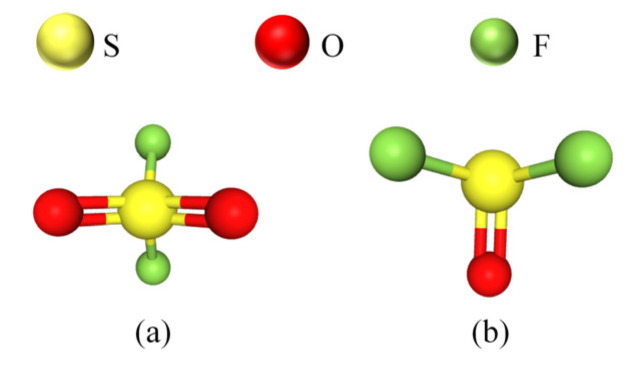
Molecular structure of (**a**) SO_2_F_2_ and (**b**) SOF_2_ [18,19].

The target gases, SO_2_F_2_ and SOF_2_, are both strongly polar substances [18,19]. Figure 3 shows their molecular structures. The tetrahedral structure and high electronegativity make SOF_2_ molecules extremely stable. SO_2_F_2_ has physicochemical properties that are similar to those of SOF_2_. However, the greater number of oxygen atoms in SO_2_F_2_ makes it extra-active. The molecular structures of SO_2_F_2_ and SOF_2_ determine the capability to transfer charge and adsorption energy with metallic media [20].

Considering the adsorption of SO_2_F_2_ and SOF_2_, metallic SUS304 and Al6061 are solid solutions filled with atoms between crystals. The adjacent atoms are in a fixed position, due to the existence of electrostatic attraction. Atoms on the inner surfaces of gas cells are in direct contact with the polar gas and have fewer adjacent atoms than those around the inner-layer atoms. To compensate for the imbalance of electrostatic attraction, it may adsorb the surrounding SOF_2_ or SO_2_F_2_ gas molecules.

For PVDF and PTFE, the adsorption effect is varied, due to their molecule structures. For example, in PVDF, the columnar structure forms a 3D network skeleton with a multi-microporous structure through the connection of fluorine atom substituents. The microporous structure provides PVDF with a higher specific surface area and stronger porosity than the metals, which indicates that PVDF would adsorb the SOF_2_ or SO_2_F_2_ polar molecules mainly through the Van der Waals force.

### 3.3. Gas Adsorption and Diffusing

**Figure 4:**
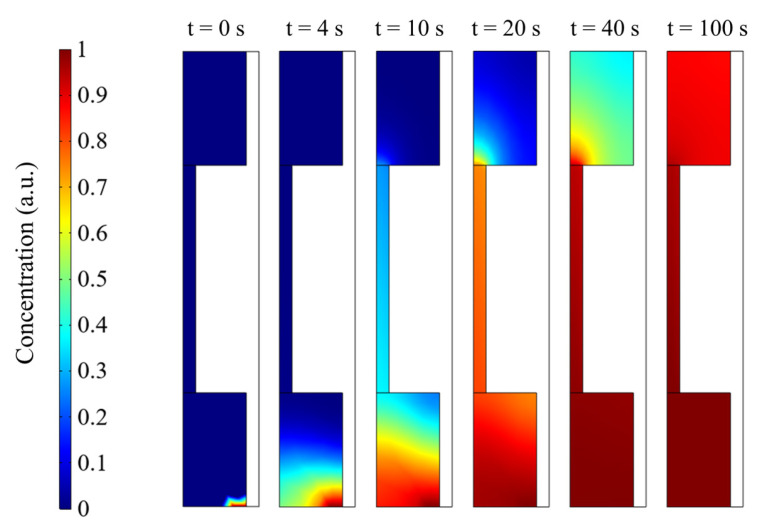
Temporal dynamic airflow and concentration distribution inside the gas cell.

By using the finite element method, a model of gas flow and adsorption in the gas cell was established. The simulation of gas adsorption and diffusion was based on the Transport of Diluted Species Interface model and the General Form Boundary Partial Differential Equations Interfaces in the software package. The effect of adsorption at the inner layer of the PAC on the gas concentration was studied and is shown in Figure 4. The inner surface of the central acoustic resonator was set as the adsorption place, for simplicity.

Figure 4 shows the temporal dynamic gas concentration inside the PAC, up to 100 s. After ventilating for 40 s, the gas concentration in the central acoustic resonator basically becomes saturated. Normally, the microphone for acoustic detection is located at the central position of the PAC, which means that the detected gas concentration would be constant after 40 s ventilation.

**Figure 5:**
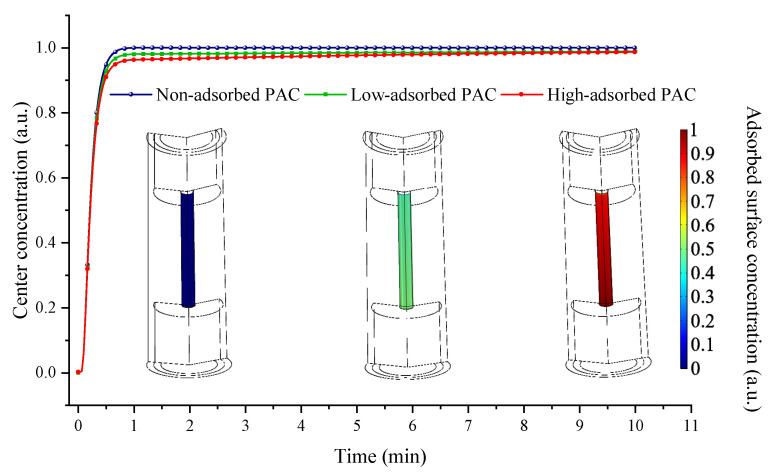
Temporal gas concentration at the center of PACs with varied absorptions.

A concentration probe was set in the center of the acoustic resonator of each PAC to monitor the change of the concentration during the gas ventilation, as shown in Figure 5. The gas concentration distribution at the inner surfaces when ventilating for 40 s is also illustrated. With the same ventilation time, the gas concentration in the PAC will decrease, due to the increased material adsorption effect. Higher adsorption leads to extended ventilation time for the gas exchange balance in the PAC.

## 4. Experiment and Discussion

### 4.1. Experimental PAS System

**Figure 6:**
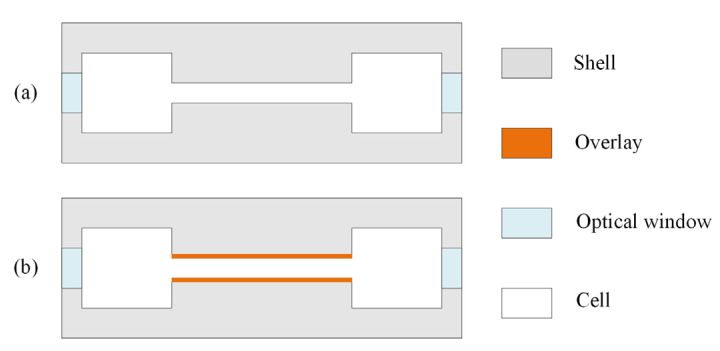
Schematic structures of PACs made of (**a**) metal or (**b**) with inner-coated plastic.

In the PAS experiment, the designed PAC was generally made by a metal-casting process. The metallic SUS304-PAC and Al6061-PAC were directly cast and assembled, as schematically shown in Figure 6a. The plastic materials, i.e., PVDF and PTFE, were sprayed and deposited on the inner walls of the metallic PACs, with an estimated thickness of about 300 microns, PVDC-PAC and PTFE-PAC, as shown in Figure 6b. The fabricated four PACs were all tested and compared in the experiments.

**Figure 7:**
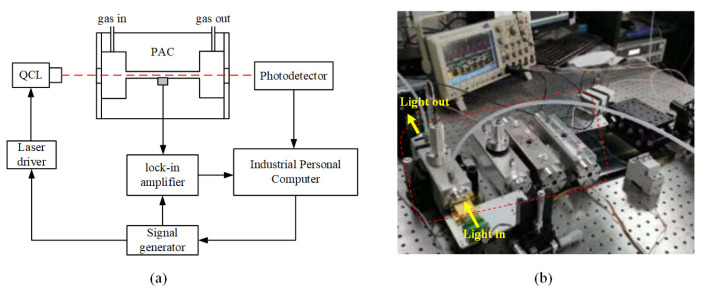
(**a**) Schematic of the PAS experimental system; (**b**) core modules including the four PACs.

The gas sensing experiment is schematically shown in Figure 7a. There were two distributed feedback quantum cascade lasers (DFB-QCL): 6648 nm (Alpes Lasers, model BG-6-7, power 250 mW) and 7463 nm (AdTech, model 7.43 μm DFB QCL, power 250 mW) for detecting SO_2_F_2_ and SOF_2_, respectively. The laser driver module included a current controller (ILX Lightwave, model LDX-3525) and a TEC temperature controller (Alpes Lasers, model TC-3), which were used to control the output wavelength of the laser. The signal generator (FLUKE, model FLUKE284) was used to generate a swept modulation signal to modulate the laser output. The microphone (BSWA TECH, model MPA201) detected the acoustic signal and converted it into a voltage signal. The voltage signal was then processed by a lock-in amplifier (Stanford, model SR830) to extract the second harmonics. The PD (VIGO, model PVI-4TE-4) was used to monitor the background light power to compensate for laser power fluctuations. Figure 7b shows the key modules of the PAS experimental system, where the four PACs used in the experiment are labeled by the dashed red box.

**Figure 8:**
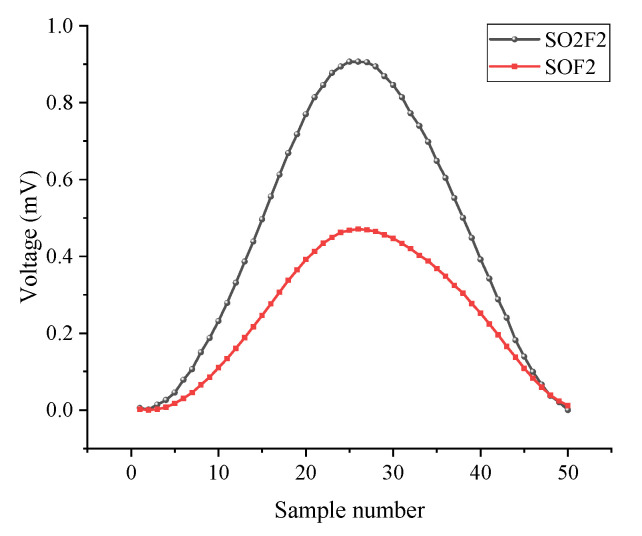
Measured second harmonics for the two gases.

SO_2_F_2_ and SOF_2_ gases were tested separately using the system. The concentrations of the used gases, which were mixed and diluted with standard concentration gases, were both 80 ppm. As acknowledged in the literature, the targets gases, i.e., SO_2_F_2_ and SOF_2_, have tens of ppm concentrations [21]. Figure 8 presents the measured second harmonic signals using the SUS304-PAC for both gases. The maximum value of this harmonic waveform in Figure 8 was used to derive the gas concentration.

### 4.2. Adsorption Analysis for SO_2_F_2_

**Figure 9:**
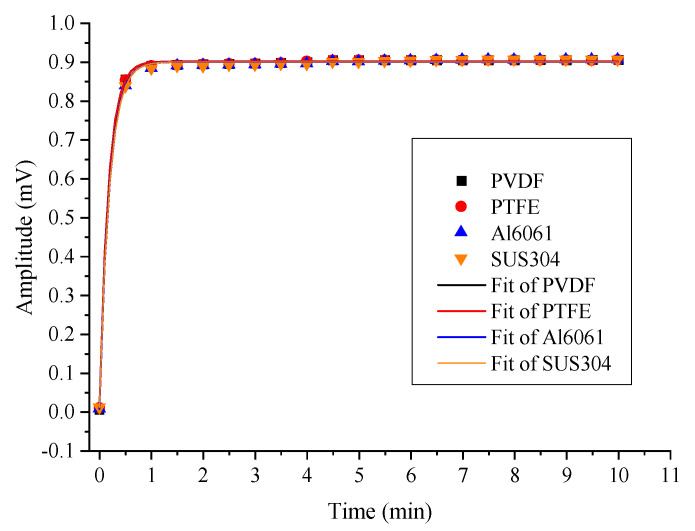
Amplitude response of four PACs for SO_2_F_2_.

Based on the second harmonic detection technique, the gas adsorption in the PAC is represented by the amplitude of the concentration-related harmonic signal. The adsorption effect of SO_2_F_2_ on the PACs made of different materials was tested and is shown in Figure 9.

The measured results were fitted with a first-order exponential function and the coefficients of determination (COD) were above 0.99. The calculated maximum value of the second harmonic amplitude, according to fitting equations and the response time to reach 90%, are presented in Table 1. Data show that the stable maximum values of PVDF-PAC, PTFE-PAC, Al6061-PAC, and SUS304-PAC were 0.9055, 0.9068, 0.9063, and 0.9065, respectively. The 90% response time was 24 s, 25 s, 27 s, and 27 s, respectively. There were slight differences (approximately 10%) in adsorption-related response times between the four PACs, while the amplitudes were basically identical.

For SO_2_F_2_ detection, PVDF and PTFE could indeed reduce the adsorption effect and shorten the response time. The maximum temporal difference between the metals and plastics was about 3 s and 10%, while the achieved magnitude was similar for these PACs.

### 4.3. Adsorption Analysis for SOF_2_

**Figure 10:**
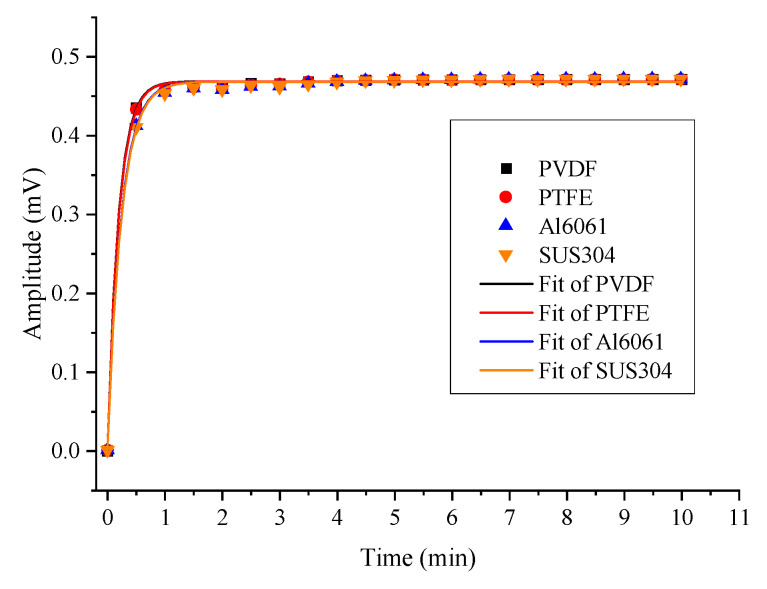
Amplitude response of four PACs for SOF_2_.

For SOF_2_, the temporal change of the second harmonic amplitude is shown in Figure 10. The measured results were fitted with a first-order exponential function with a COD above 0.99. The calculated data are included in Table 2. The results show that the maximum values were 0.47076, 0.47092, 0.47112, and 0.47116, and the response times to reach 90% were 28 s, 28 s, 34 s, and 35 s, respectively. The temporal response difference between the metals and plastics was about 6s and 20%.

It is to be noted that metals usually behave with advanced casting capability, thermal conductivity, and external noise isolation, which are also essential for practical PAS-based gas sensors. By using the SUS304 photoacoustic gas cell, the detailed determination of the concentration of adsorbed substances could also be found in our recent publication [22].

## 5. Conclusion

In this paper, optical gas-cell dynamic adsorption with different materials in the PAS-based gas sensor for SOF_2_ and SO_2_F_2_ were studied. The adsorption characteristics and photoacoustic excitation effects of SUS304, Al6061, PVDF, and PTFE were theoretically analyzed and experimentally tested. Inner coating with PVDF or PTFE could reduce the polar adsorption effects of SO_2_F_2_ and SOF_2_, compared with those of Al6061 and SUS304. It could shorten the response time required for the stabilized concentration by about 10% and 20%, respectively. The achieved signal amplitudes were identical for the different PACs, which indicated constant sensing accuracy. In practical applications, in addition to the material adsorption of SO_2_F_2_ and SOF_2_, the gas ventilation, thermal conduction, and fabrication capability should be comprehensively considered. This work provides guidance for optical gas-cell material selection for PAS gas sensing in practical applications.

## Figures and Tables

**Table 1:** Measured response of four PACs for SO_2_F_2_.

Materials	Max Value	90% Max	90% Response Time
PVDF	0.9055	0.81495	24
PTFE	0.9058	0.81522	25
Al6061	0.9063	0.81567	27
SUS304	0.9065	0.81585	27

**Table 2:** Measured response of four PACs for SOF_2_.

Materials	Max Value	90% Max	90% Response Time
PVDF	0.47076	0.42368	28
PTFE	0.47092	0.42383	28
Al6061	0.47112	0.42347	34
SUS304	0.47116	0.42404	35

## Data Availability

The datasets generated during the current study are available from the corresponding author on reasonable request.
